# Post-Progression Survival after EGFR-TKI for Advanced Non-Small Cell Lung Cancer Harboring EGFR Mutations

**DOI:** 10.1371/journal.pone.0135393

**Published:** 2015-08-11

**Authors:** Yoshihito Kogure, Hideo Saka, Masahide Oki, Toshiki I. Saito, Shimaa Nour Moursi Ahmed, Chiyoe Kitagawa, Kazuyoshi Imaizumi

**Affiliations:** 1 Department of Respiratory Medicine, National Hospital Organization Nagoya Medical Center, Nagoya, Aichi, Japan; 2 Clinical Research Center, National Hospital Organization Nagoya Medical Center, Nagoya, Aichi, Japan; 3 Department of Respiratory Medicine, Fujita Health University School of Medicine, Toyoake, Aichi, Japan; University of Barcelona, SPAIN

## Abstract

**Background:**

Non-small cell lung cancer (NSCLC) patients that harbor epidermal growth factor receptor (EGFR) mutations benefit from receiving an EGFR-tyrosine kinase inhibitor (TKI); however, post-progression survival (PPS) after EGFR-TKI treatment has not been sufficiently studied.

**Methods:**

We retrospectively reviewed the clinical data from stage IV or recurrent NSCLC patients who harbored EGFR mutations and who received EGFR-TKI as their first-line treatment in our institute between 2009 and 2011.

**Results:**

In total, 36 patients received EGFR-TKI treatment as their first-line therapy. Of those 36 patients, 30 experienced recurrence and were enrolled in this study. The median progression-free survival (PFS) of these patients was 8.2 months. Twelve patients received EGFR-TKI treatment beyond the diagnosis of progressive disease (PD), and 8 received second-line therapy. The PPS after EGFR-TKI treatment was 9.1 months, and survival after the termination of EGFR-TKI treatment in those patients treated with second-line chemotherapy was 13.9 months. The site of relapse was investigated and PFS in EGFR-TKI-treated patients with relapse in the brain (11.6 months) showed a trend toward a longer PFS compared with patients with relapse at other sites (8.2 months). The median PPS after EGFR-TKI treatment also showed the same trend in each group (12.9 and 9.2 months, respectively).

**Conclusions:**

The PPS after EGFR-TKI treatment failure was 9.1 months, while the survival of patients who underwent second-line chemotherapy after the termination of EGFR-TKI treatment was 13.9 months, comparable with the overall survival of EGFR mutation-negative patients, as previously reported. The prognosis of these NSCLC patients with EGFR mutations varied according to the sites of recurrence after first-line EGFR-TKI treatment. Of particular note was the prognosis of patients with brain metastases, which tended to be better than that of patients with metastases to other sites.

## Introduction

In previous reports, advanced non-small cell lung cancer (NSCLC) patients harboring epidermal growth factor receptor (EGFR) mutations received an EGFR-tyrosine kinase inhibitor (TKI) and achieved a progression-free survival (PFS) of 10 months and an overall survival (OS) of 24 months [1–5]. This was a markedly improved prognosis when compared with the reported OS of approximately 14 months that was achieved in EGFR mutation-negative patients who received treatment with a cytotoxic anti-cancer agent [6, 7].

Post-progression survival (PPS) is defined as the survival time with progressive disease (PD) following primary treatment [8]. PPS can be estimated by calculating the difference between OS and PFS. PPS after EGFR-TKI treatment is estimated to be approximately 14 months, but there are currently no published data on PPS after EGFR-TKI treatment. In patients with advanced NSCLC for which chemotherapy is indicated, it is not known whether the OS in EGFR mutation-negative patients is equivalent to PPS of EGFR mutation-positive patients after EGFR-TKI treatment failure. In the present study, PPS after EGFR-TKI treatment failure and the treatments following EGFR-TKI treatment were investigated.

## Materials and Methods

### Subjects

Data were collected retrospectively from medical records. The patients had stage IIIB/IV or recurrent NSCLC with active EGFR mutations, an ECOG performance status (PS) of 0–3, and received EGFR-TKI treatment in our institute. The study was conducted between January 2009 and December 2011. In routine clinical practice, EGFR-TKI treatment is often continued until clinical PD beyond the Response Evaluation Criteria in Solid Tumors (RECIST) PD. In the present study, PD was not based on RECIST ver. 1.1, but on the clinical judgment of the attending physician.

This study was approved by the institutional review board of the Nagoya Medical Center on February 19, 2014 (approval number: 2013–722). No informed consent was obtained because this study was an observational study. Patient records were anonymized and de-identified prior to analysis. PFS and OS were measured from the date of starting EGFR-TKI treatment. Survival after EGFR-TKI treatment termination was measured from the date of termination of treatment with EGFR-TKI.

### Statistical Methods

PFS, PPS, and OS were assessed using the Kaplan-Meier method and compared using the log-rank test. Analyses were conducted using PASW Statistics 21.0 (SPSS Inc., Chicago, IL, USA).

## Results

**Fig 1:**
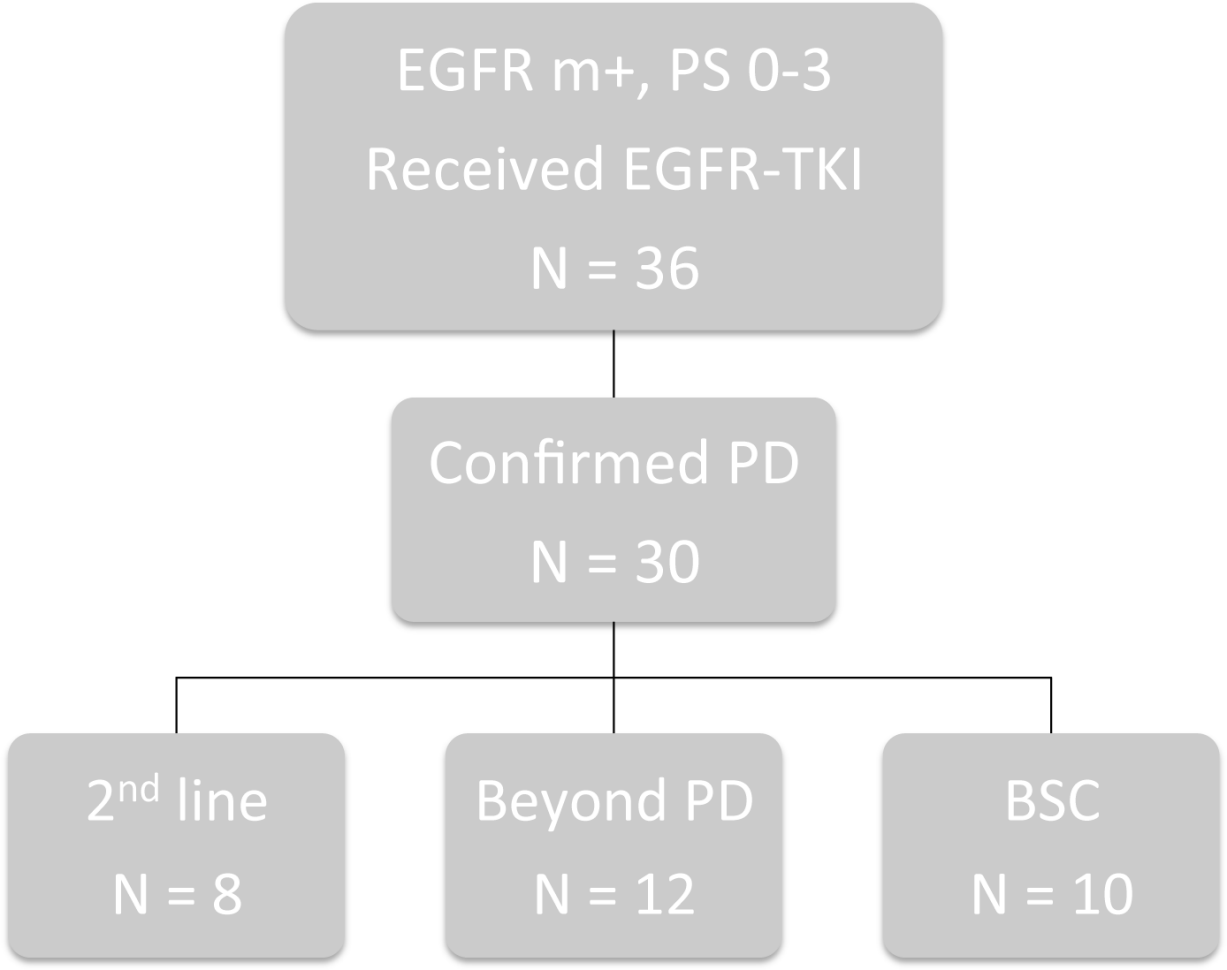
Patient Profile. EGFR, epidermal growth factor receptor; m+, mutation-positive; PD, progressive disease; BSC, best supportive care.

Thirty patients who initially received EGFR-TKI treatment and showed PD between January 2009 and December 2011 were included (Fig 1), and their characteristics are shown in Table 1.

**Table 1:** Patient characteristics.

	N
**Male/Female**	4/26
**Age, median (range)**	76 (38–97)
**EGFR-TKI** Gefitinib/Erlotinib	29/1
**Relapse site** brain/pleural effusion/bone/lung	5/13/6/8
**ECOG PS on PD** 0/1/2/3/4	2/16/6/5/1
**Sequential therapy** second-line/beyond PD/BSC	8/12/10
**Second-line therapy (n = 10)** doublet/mono	6/2

Abbreviations: EGFR-TKI, epidermal growth factor receptor-tyrosine kinase inhibitor; ECOG, Eastern Cooperative Oncology Group; PS, performance status; PD, progressive disease; BSC, best supportive care

**Fig 2:**
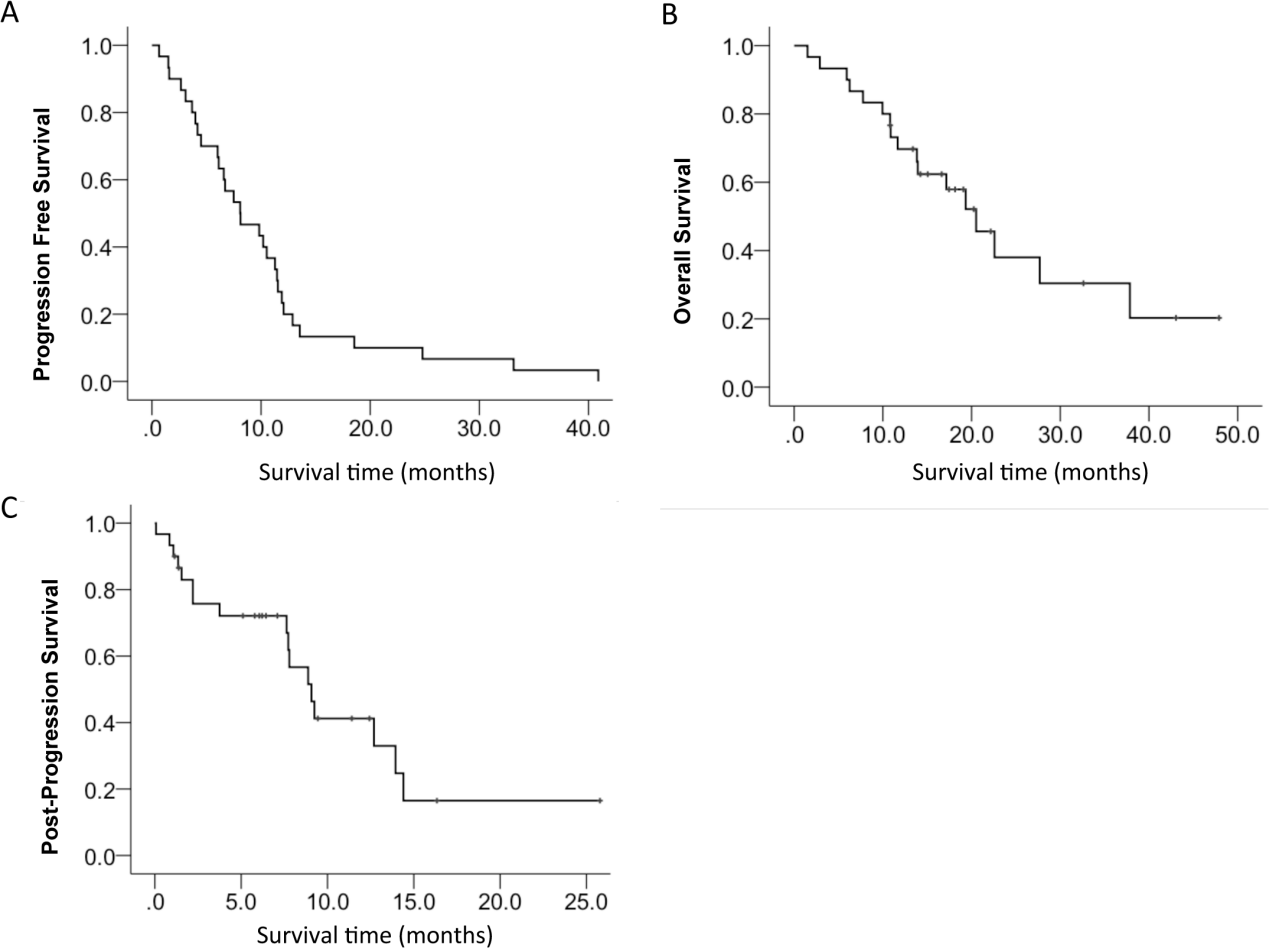
Kaplan-Meier Plot. A, progression-free survival (PFS); B, overall survival (OS); and C, post-progression survival (PPS).

The majority of the patients were women (86.7%) and the median age was 76 years (range: 38–97). Only 1 patient received erlotinib as their EGFR-TKI, while the others received gefitinib. The most common site of initial recurrence was pleural effusion (13 cases), followed by the lung (8 cases), bone (6 cases), and brain (5 cases, including multiple recurrences). The most common ECOG PS at the time of PD with EGFR-TKI treatment was 1 (16 patients). After PD, 8 patients underwent second-line therapy, 12 continued EGFR-TKI treatment, and 10 received best supportive care (BSC). All 12 of the patients who received EGFR-TKI treatment after their initial PD continued treatment without taking a EGFR-TKI treatment holiday. Among the patients who underwent second-line therapy, 6 patients received carboplatin+pemetrexed, 1 patient received S-1, and 1 patient received pemetrexed. The median PFS in all patients was 8.1 months (range: 3.9–12.3; Fig 2A), while the median OS was 20.5 months (range: 14.8–26.3; Fig 2B). The median PPS was 9.1 months (range: 7.1–11.0; Fig 2C), which was slightly lower than the estimated PPS of 12.4 months.

**Fig 3:**
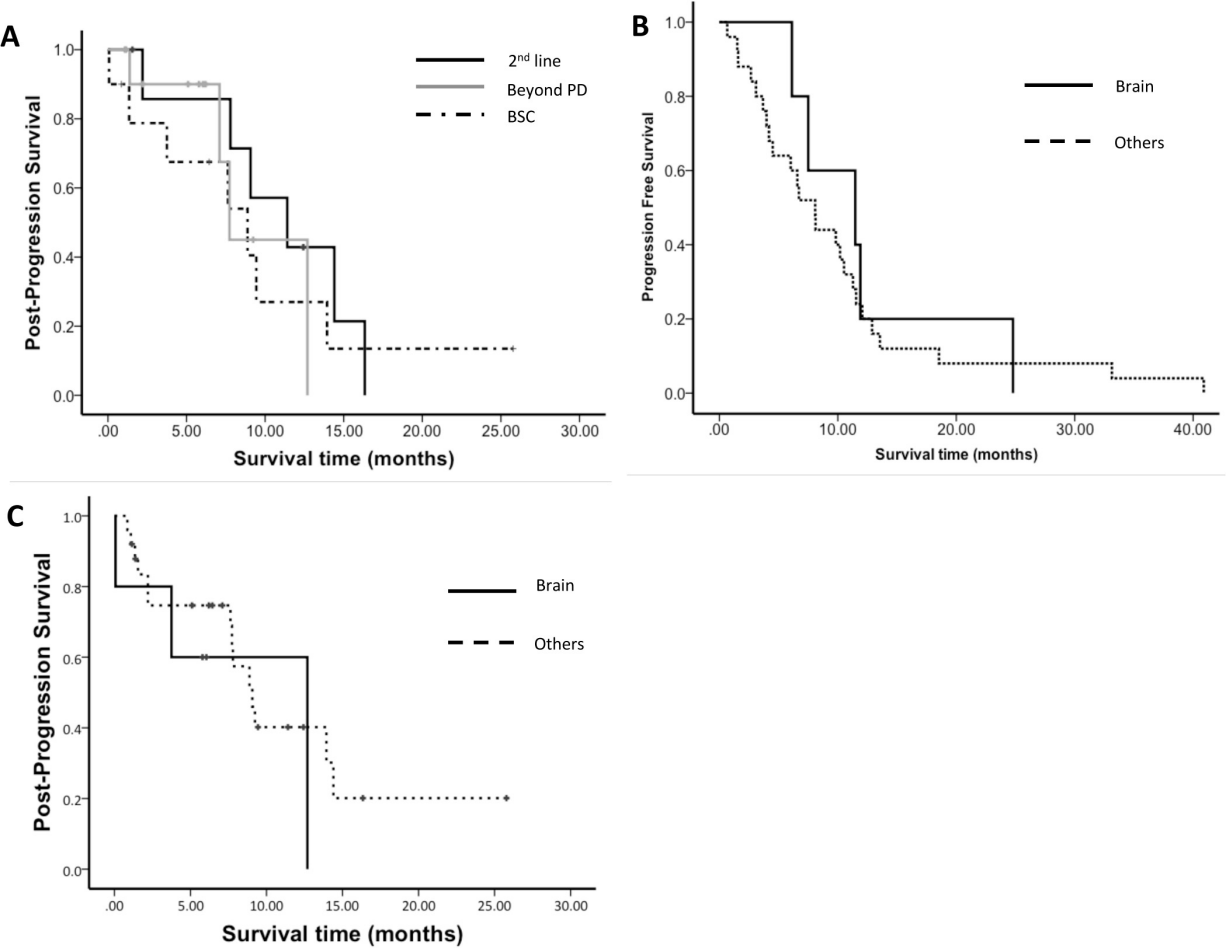
Kaplan-Meier Plot. **A,** Post-progression survival (PPS) according to sequential therapy; B, Progression-free survival (PFS) in EGFR-TKI-treated patients by relapse site.; C, PPS according to relapse site. PD, progressive disease; BSC, best supportive care.

**Fig 4:**
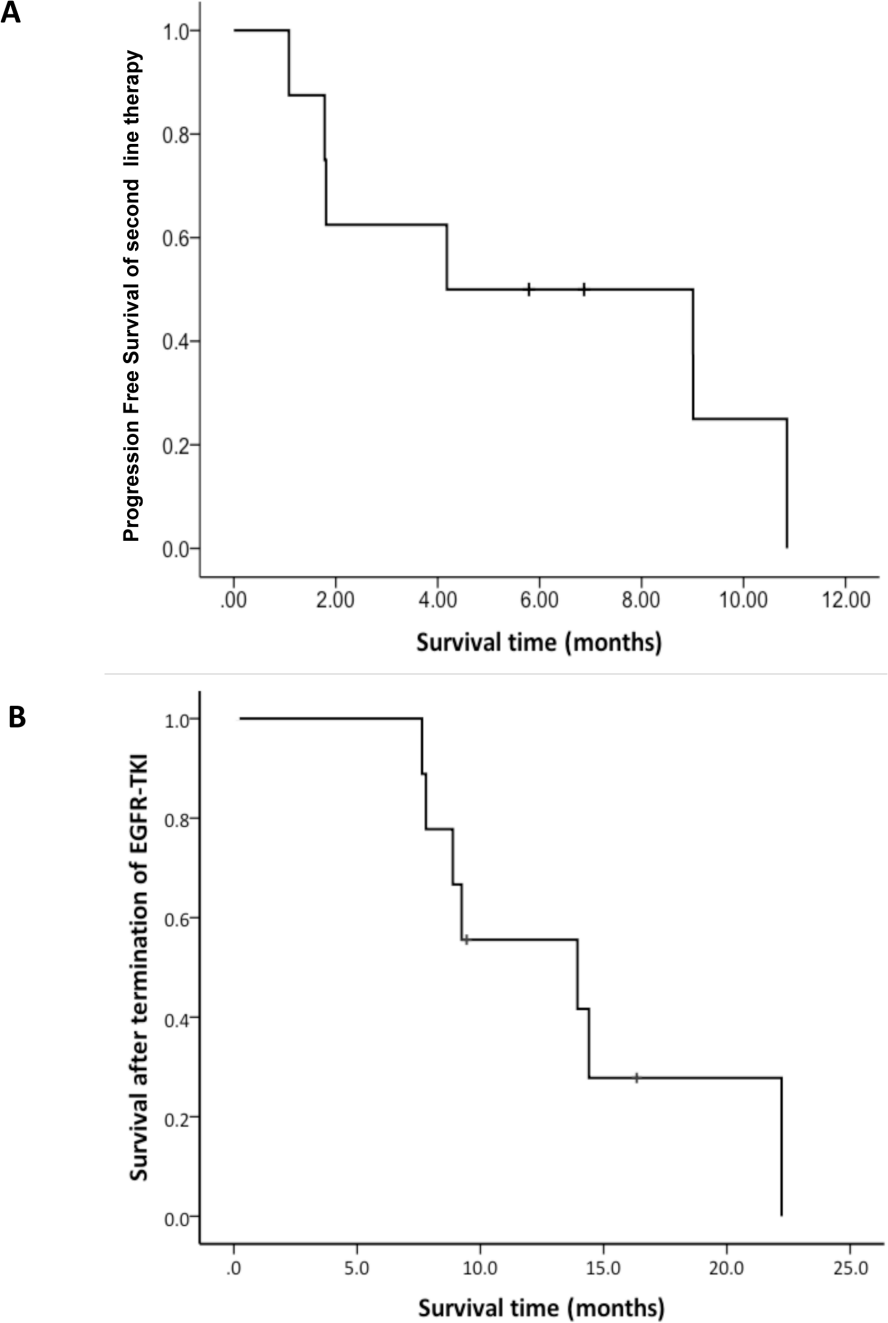
Kaplan-Meier Plot. A, PFS of the patients who received second-line therapy; B, Survival time after termination of EGFR-TKI treatment among patients who received second-line chemotherapy. PFS, progression-free survival.

The PPS of the patients was analyzed based on subsequent treatment (Fig 3A) and demonstrated improvements in the second-line therapy (11.4 months) as compared with those of all subjects. The PFS of the patients who received second-line therapy was 3.4 months (range: 0.0–10.8; Fig 4A). One of 5 patients received chemotherapy after progression of EGFR-TKI treatment beyond PD. In total, 9 patients received chemotherapy after termination of EGFR-TKI treatment and their median survival time was 13.9 months (range: 2.5–24.4; Fig 4B).

**Fig 5:**
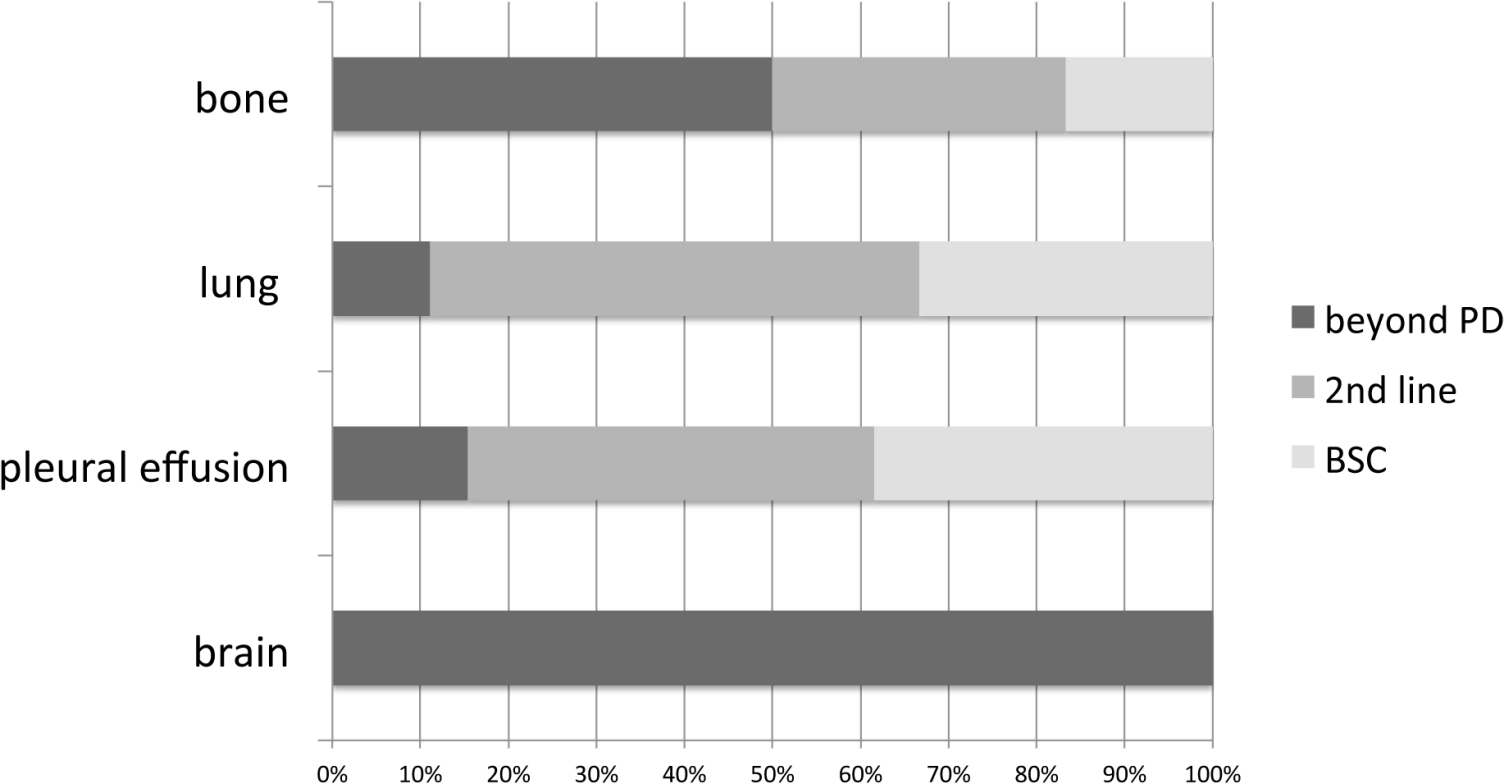
Sequential Therapy According to Relapse Site.

The post-progression subsequent treatment was examined by the site of recurrence. All patients with brain metastases continued EGFR-TKI treatment. Most patients with pulmonary and pleural effusion recurrences were switched to different anti-cancer agents (Fig 5). The PFS was examined by the site of recurrence (Fig 3B). The PFS of the brain metastases group (11.5 months, range: 2.9–20.0) was longer than that of the recurrence group (8.1 months, range: 5.6–10.6), although no significant difference was noted (p = 0.605). PPS at the site of recurrence showed a similar trend: 12.7 months for the brain metastases group and 9.1 months for the other groups (p = 0.465; Fig 3C).

## Discussion

Previous phase III studies were conducted to compare PFS between EGFR-TKI and an anti-cancer agent as first-line therapy for EGFR mutated NSCLC patients. The present study focused on post-treatment PPS for the first time. In the present study, PFS (8.1 months) and OS (20.5 months) were shorter than those previously reported with EGFR-TKI as the initial treatment (PFS: 10 months, OS: 24 months) [1–5], but were within the expected range considering that they were extracted from the database of routine clinical practice, where patients with a poor PS and those with organ dysfunction are mixed (unlike in clinical studies). Also, the PPS (9.1 months) was relatively shorter.

EGFR-TKI treatment is only effective for EGFR mutation-positive patients. Thus, therapeutic strategies and prognoses differ depending on the presence or absence of EGFR mutation. Of note, the positive rate of EGFR mutation in East Asia (30%), including Japan, is higher than that in Europe and the United States (8%) [9–11]. Phase III comparative studies, using OS as a primary endpoint, have subdivided patients into EGRF-positive and-negative groups, and consequently lowered the number of cases accumulated and limited the analyses or conclusions that could be drawn from the data.

The OS of advanced NSCLC EGFR mutation-negative patients in Japan is approximately 14 months [6, 7]. The survival time of EGFR mutation-positive patients who received second-line chemotherapy after EGFR-TKI treatment termination was 13.9 months, as shown in the present study. Thus, the OS of EGFR mutation-negative patients was equivalent to the survival time of EGFR mutation-positive patients. If the OS of EGFR mutation-negative patients is equivalent to the survival time of EGFR mutation-positive patients, the quality of the study may be maintained even if EGFR mutation-positive patients with EGFR-TKI treatment failure are included in a conventional clinical study of EGFR mutation-negative patients. Planning a clinical study with such eligibility criteria would accelerate the completion of the study as compared with a conventional study that included only EGFR mutation-negative patients.

The recurrence rate of brain metastases is reported to be low in patients receiving EGFR-TKI treatment [12, 13]. EGFR-TKI barely passes through the blood-brain barrier [14, 15]; thus, the effects of EGFR-TKI treatment on brain metastases remain controversial. In the present study, 4 of 5 patients with brain metastases after EGFR-TKI treatment underwent radiotherapy for the brain metastases, and all continued EGFR-TKI treatment. PFS tended to be longer in patients with brain metastases than in those with recurrence in other sites. In NSCLC patients with focal progression after EGFR-TKI treatment, EGFR-TKI treatment was continued along with local treatment, providing long-term disease control [16]. These results suggest that even in patients with brain metastases, for which EGFR-TKI treatment is less effective, survival can be extended by continuing EGFR-TKI treatment after radiotherapy as long as EGFR-TKI treatment continues to be effective in extra-cranial lesions.

Recently, Nishie et al. [17] and Watanabe et al. [18] reported the effectiveness of EGFR-TKI treatment that continued even after EGFR-TKI treatment failure (so-called beyond PD). Watanabe et al. did not describe the sites of recurrence in cases of EGFR-TKI treatment failure, but Nishie et al. reported that, as a result of multivariate analysis, brain metastases were not correlated with survival. In the present study, only patients with recurrence in intra-cranial lesions underwent treatment beyond PD after EGFR-TKI treatment failure. This suggests that the decision of whether or not to continue EGFR-TKI treatment in routine clinical practice is determined based on the site of recurrence after EGFR-TKI treatment failure. In the future, in planning a clinical study on the effects of EGFR-PKI treatment beyond PD after first-line EGFR-TKI treatment failure, we should collect data on the sites of recurrence to examine whether the sites correlate with the efficacy of EGFR-TKI treatment beyond PD.

This retrospective study with varying imaging periods was limited by the fact that PFS could not be accurately determined. In addition, the subsequent treatment was not specified.

## Conclusions

The PPS and OS results in this study should be confirmed by a prospective or cohort study to increase the ability to accumulate cases in future clinical studies. Prognoses varied with the sites of recurrence after first-line EGFR-TKI treatment. Of note, the prognoses of patients with brain metastases tended to be better than in those with metastases to other sites. These results would facilitate the development of therapeutic strategies beyond PD diagnosis after first-line EGFR-TKI treatment failure.
